# Targeting Genome Integrity in *Mycobacterium Tuberculosis*: From Nucleotide Synthesis to DNA Replication and Repair

**DOI:** 10.3390/molecules25051205

**Published:** 2020-03-07

**Authors:** Riccardo Miggiano, Castrese Morrone, Franca Rossi, Menico Rizzi

**Affiliations:** Department of Pharmaceutical Sciences, University of Piemonte Orientale, Via Bovio 6, 28100 Novara, Italy; castrese.morrone@uniupo.it (C.M.); franca.rossi@uniupo.it (F.R.)

**Keywords:** *Mycobacterium tuberculosis*, nucleotide synthesis, DNA replication, DNA repair, antitubercular drugs, novel drug targets

## Abstract

*Mycobacterium tuberculosis* (MTB) is the causative agent of tuberculosis (TB), an ancient disease which still today causes 1.4 million deaths worldwide per year. Long-term, multi-agent anti-tubercular regimens can lead to the anticipated non-compliance of the patient and increased drug toxicity, which in turn can contribute to the emergence of drug-resistant MTB strains that are not susceptible to first- and second-line available drugs. Hence, there is an urgent need for innovative antitubercular drugs and vaccines. A number of biochemical processes are required to maintain the correct homeostasis of DNA metabolism in all organisms. Here we focused on reviewing our current knowledge and understanding of biochemical and structural aspects of relevance for drug discovery, for some such processes in MTB, and particularly DNA synthesis, synthesis of its nucleotide precursors, and processes that guarantee DNA integrity and genome stability. Overall, the area of drug discovery in DNA metabolism appears very much alive, rich of investigations and promising with respect to new antitubercular drug candidates. However, the complexity of molecular events that occur in DNA metabolic processes requires an accurate characterization of mechanistic details in order to avoid major flaws, and therefore the failure, of drug discovery approaches targeting genome integrity.

## 1. Introduction

According to the most recent Global Tuberculosis Report (2019) edited by the World Health Organization (WHO), 10 million new Tuberculosis (TB) cases worldwide were estimated in 2018; moreover, there were 1.2 million TB deaths amongst HIV-negative patients, and an additional 250,000 deaths amongst HIV-positive patients [1]. The global control and management of TB is facing the worsening scenarios of the spreading of multi-drug resistant (MDR) MTB strains, mostly referred to as rifampicin-resistant TB, as well as extensively-drug resistant (XDR) MTB strains that are resistant to first- and second-line anti-tubercular drugs [2]. In addition to drug resistance events, the adherence to long-term antitubercular therapy, the hepatotoxicity of most currently used drugs and reduced drug tolerance [3,4] all strongly support the development of new therapies for both drug-sensitive and drug-resistant TB with novel mechanisms of action. The genotypic information derived from MTB genome sequencing [5] guides the current anti-TB pipeline for which new drugs (bedaquiline and delamanid) have recently been approved, and several candidates entered Phase II and Phase III clinical trials [6]. However, for a definitive solution to the clinical management of drug-resistant tuberculosis, other innovative drugs are urgently needed.

The criteria for developing new anti-TB drug candidates are well established [7,8,9,10]. In addition to a low toxicity level and reduced adverse reactions, there are a number of other factors that any promising anti-TB molecule candidate should fulfil: (i) to be more potent than those currently available, with the aim of shortening the duration of treatment; (ii) to be active on new targets in order to be effective on MDR-TB and XDR-TB; (iii) to be compatible with antiretroviral therapy, as many patients are co-infected with HIV; and (iv) to show no antagonism to other TB drugs or drug candidates so that a multi-component regimen can be applied. Considering the unique feature of the MTB cell envelope—being mainly built on the mycolylarabinogalactan-peptidoglycan complex, which connects the peptidoglycan to the mycobacterial outer membrane—a number of antitubercular drugs target the synthetic pathway of key components of the cell envelope. Among these, the frontline agents isoniazide and ethambutol constitute the major TB drug arsenal active on cell wall biogenesis, together with the second line agent d-cycloserine. More recently, other drugs or drug candidates have been developed that act on new targets, including PBTZ169, targeting ecaprenylphospho-β-d-ribofuranose 2-oxidase [11], and BM212 and analogs inhibiting mycobacterial membrane protein Large 3 [12] and benzofuran TAM16, active on polyketide synthase Pks13 [13]. Alternative validated target pathways include DNA transcription, which is inhibited by rifampicin, protein synthesis, which is blocked by oxazolidinones [14], ATP synthesis by Q203 (acting on cytochrome bc1 complex) [15] and Bedaquiline, which inhibits F_0_F_1_ATP synthase [16].

Despite its central role in cell development and function, a limited number of approved TB drugs target DNA metabolism, which includes all the reactions involved in DNA replication and repair. Indeed, these have been limited exclusively to the fluoroquinolones that interfere with DNA gyrase and DNA topoisomerase activity, and are frequently used as second-line drugs for the treatment of MDR-TB. However, fluoroquinolones’ capability in reducing the duration of therapy in murine models of TB is strengthening their use as first-line drugs [17]. Several reviews have been published recently that focus on the current regimens and emerging drugs acting on the key cellular process of DNA metabolism in pathogenic bacteria; a number of excellent works investigate the DNA replication and repair pathways as sources of molecular targets [18,19,20,21], as well as the biosynthesis of nucleic acids precursors—the nucleotides [22]. The present review gives a concise overview of our current knowledge and understanding of key processes involved in genome maintenance in MTB, particularly on purines and pyrimidine biosynthesis, DNA replication and DNA repair, with the aim to highlight their potential impact for the development of novel drugs against TB. Particular emphasis will be placed on recent results concerning the phenotypic screenings and biochemical characterization of macromolecular complexes acting in DNA metabolic pathways, as well as the innovative strategies that have been proposed to interfere with the formation of such complexes as potential sources of new targets for TB drug development.

## 2. Targeting Purine and Pyrimidine Ribonucleotide Synthesis

### 2.1. Purine Biosynthesis

**Figure 1:**
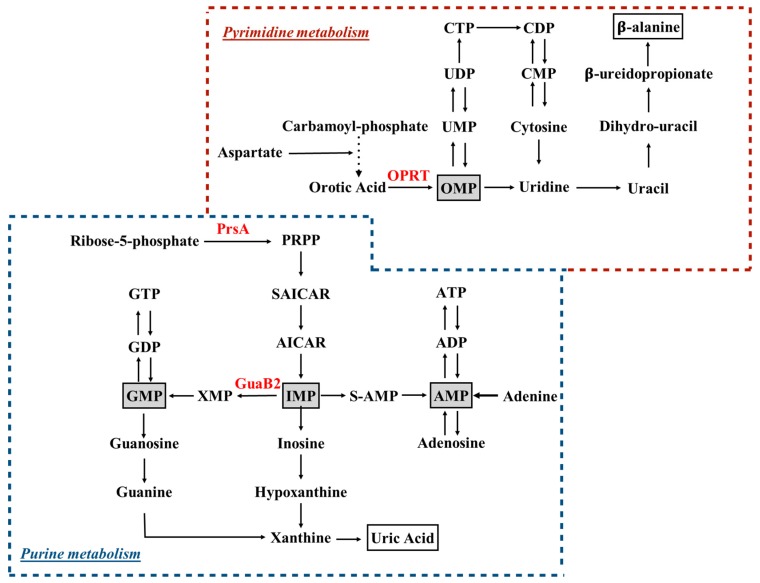
Schematic overview of purine and pyrimidine metabolism. Ribose-5-phosphate and carbamoyl-phosphate are the starting points of purine and pyrimidine biosynthetic pathways, respectively. Key intermediates, across de novo biosynthesis and salvage pathways, are highlighted in gray boxes. End-products of purine and pyrimidine catabolism (i.e., uric acid and β-alanine) are in white boxes. Enzymes discussed in the manuscript are depicted in red. PRPP, 5-phosphorybosyl-1-pyrophosphate; OPRT, orotate phosphoribosyltransfrase, PrsA, PRPP synthetase; S-AMP, adenylosuccinate; SAICAR: succinylaminoimidazole carboxamide ribotide.

Purine nucleotides are of key importance in living organisms, since they provide the building blocks for DNA and RNA. Two main pathways exist in most organisms, including mycobacteria, to which from here onward we specifically refer for the production of these compounds: the de novo biosynthetic pathway (Figure 1), in which nucleotides are synthesized from 5-phosphoribosylpyrophosphate (PRPP) in a multi enzymatic process, and the salvage pathways, in which nucleotides are retrieved after the catabolism of nucleic acids and coenzymes.

In mycobacteria, the first half of the de novo purine biosynthetic pathway involves the generation of an aminoimidazole moiety attached to a ribose; the series of reactions starts from PRPP synthetized by phosphoribosylpyrophosphate synthetase [23,24,25], whose enzymatic activity has been shown to strongly depend on inorganic phosphate, another key molecule for MTB survival [26,27]. In the second half of the pathway, the carbon atoms at positions 4 and 5 of the imidazole moiety are modified, allowing the final cyclization reaction that generates inosine 5′-monophosphate (IMP) [28]. The conversion of IMP to guanosine 5′-monophosphate (GMP) begins with the NAD^+^-dependent oxidation of IMP to xanthosine 5′-monophosphate by IMP dehydrogenase (IMPDH, GuaB), which was deeply explored as a drug target. MTB encodes three apparent homologues of IMPDH on its chromosome (*guaB1*, *guaB2* and *guaB3*), but only *guaB2* (Rv3411c) was found to encode a functional IMPDH enzyme (GuaB2) [29,30]. GuaB2 has a key role in guanine nucleotide metabolism, since it is a rate-limiting enzyme of the cascade, and its inhibition results in a depletion of cellular guanine nucleotides [31]. Usha and co-workers identified diphenyl urea-based derivatives as inhibitors of MTB GuaB2 with potent antimycobacterial activity, with the most potent compounds, DPU-2 and DPU-3, behaving as noncompetitive and uncompetitive inhibitors with respect to IMP, respectively [29]. The peculiar mechanism of inhibition sustains the potential species-specificity of such molecules avoiding cross-reactivity with the human enzyme, which shares 41% of sequence identity with the mycobacterial enzyme [32]. Further inhibition studies identified a novel series of triazole-linked mycophenolic adenine inhibitors as NAD cofactor mimics; the activity of these inhibitors was tested against both human IMPDH isoforms and against MTB GuaB2, and showed no selectivity for the bacterial enzyme [33]. Taking into account the difficulties of translating the products of target-based drug discovery into hits that show antibacterial activity, a number of target-agnostic approaches were developed based on whole-cell screenings, which select an initial set of hit compounds based on their capability to inhibit bacterial growth [34]. The drug discovery potential of such methods, namely phenotypic screening, is further reinforced by the whole genome sequencing of resistant mutants in order to identify SNPs, which suggests the potential target(s) of tested compounds are subsequently genetically and pharmacologically validated [35]. This empiric approach guided the research pipeline of two independent groups, which identified a new class of lead compound that targets MTB GuaB2 with promising antitubercular activity and limited mammalian cell toxicity [36,37]. In particular, Park et al. characterized a number of indazole sulfonamides that showed micromolar potency against MTB, behaving as uncompetitive inhibitors of the enzyme that were only effective against replicating MTB cells [36]. Amongst them, the most powerful, N-(1H-indazol-6-yl)-3,5-dimethyl-1H-pyrazole-4-sulfonamide, showed a Ki of 0.22 μM and an MIC of 2 μM. On the other hand, a parallel phenotypic screening of a library with thousands of compounds allowed the identification of the VCC234718 molecule (5-(4-cyclohexanecarbonylpiperazine-1-sulfonyl)isoquinoline, belonging to the class of sulfonamides), which inhibits MTB GuaB2 with a Ki of 0.1 μM and a uncompetitive mechanism with respect to the IMP and NAD^+^ cofactors; this was entirely characterized through structural and biochemical investigations. Moreover, VCC234718 displayed a MIC90 value of 2 μM [37]. Interestingly, both compounds, which were discovered independently by the two research teams, target the same enzyme, belong to the same chemical class and act with the same inhibitory mechanism by binding the enzyme active site in a comparable manner, and being recognized by the same molecular determinants as clearly showed by the X-ray structures. Moreover, the structural analysis of the enzyme-inhibitor complexes also explained the remarkable selectivity shown by the compounds toward the MTB GuaB2 versus the human enzyme (with a selectivity index higher than 30)—a very relevant aspect when targeting an enzyme that is also present in humans [36,37]. Finally, the structural studies clearly demonstrated why the mutation that features the Guab2 variant (Y487C) conferring MTB resistance to VCC234718 results in a still-active enzyme, which is, however, insensitive to the inhibitor action [36,37]. This observation is of great value to already start the structure-based rational design of VCC234718 derivatives, which could overcome a possible emergence of resistance.

The validation of GuaB2 as a new TB drug target stimulated further studies aiming at the discovery of hit compounds with bactericidal activity [38,39]; indeed, on the basis of previous published results [40,41,42,43,44], an expansion of the structure–activity relationship of benzoxazole-based derivatives revealed potent inhibitors of MTB GuaB2 displaying a Ki of 14 nM, though with a moderate antibacterial activity [39].

### 2.2. Pyrimidine Biosynthesis

The de novo synthesis of pyrimidine nucleotides (Figure 1) involves the coordination of a multi-enzymatic cascade in which six catalyzed reactions culminate in the formation of uridine monophosphate (UMP), the precursor of all pyrimidine nucleotides [28]. In MTB, the genes encoding five of the enzymes acting in the pyrimidine synthesis map on the *pyr* operon, and some of these genes are essential for the growth of MTB in vitro [45]. However, biochemical insights on pyrimidine biosynthesis in MTB derive from investigations performed on other bacteria. The fifth step in the pathway, which is catalyzed by orotate phosphoribosyltransfrase (OPRT) [46,47]—a type I PRTase that converts orotate to orotidine 5′-monophosphate (OMP)—has been investigated as a potential drug target, and submicromolar pyrimidin-2(1H)-one-based inhibitors of the MTB OPRT enzyme have been identified, but as yet have not been tested on MTB to evaluate antibacterial activity [48]. As reported in the introduction, both purine and pyrimidine biosynthesis strongly rely on PRPP availability, and therefore the PRPP synthetase is another attractive target for the development of antibacterial agents. In particular, MTB PRPP synthetase (PrsA, encoded by the *prsA* gene) has been validated as a robust target, and extensively biochemically and structurally characterized [49,50,51]. Therefore, at least three enzymes—namely PRPP synthase (PrsA), IMP dehydrogenase (GuaB2) and orotate phosphoribosyltransferase (PyrE), whose crystal structures has been deposited and are available for further structure-based drug design [23,36,37,47]—are now considered as strong targets, and are being investigated for the development of novel drugs against TB.

Although the current review does not focus and therefore does not detail the synthesis and maintenance of deoxynucleotide triphosphates (dNTPs), such processes are of course of major relevance for a correct DNA homeostasis, and have been targeted in drug discovery events against MTB with, amongst others, investigations on thymidylate synthase [52,53] and dUTPase superfamily enzymes [54].

## 3. Targeting MTB DNA Replication

**Figure 2:**
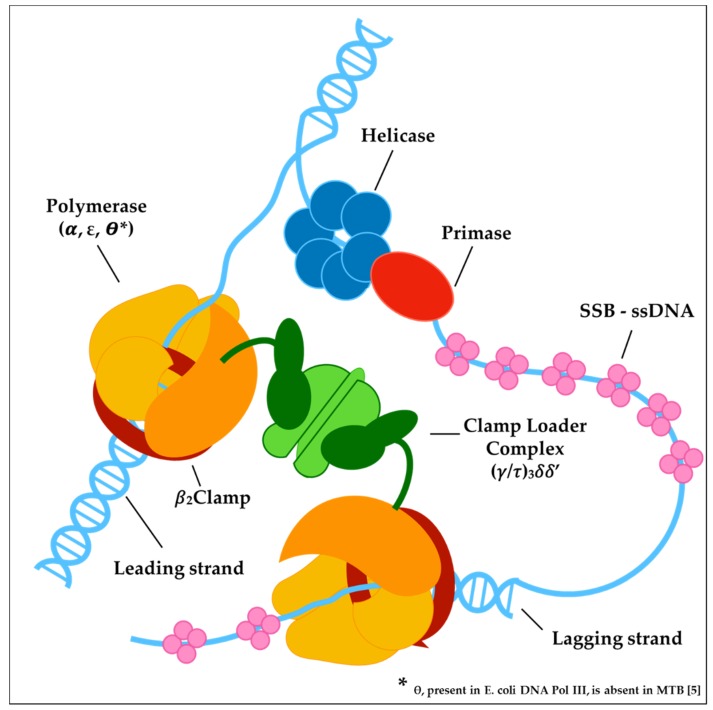
Macromolecular complexes assembled on the DNA at the replication fork. Helicase-primase complex constitutes the so-called primosome that binds the lagging strand DNA, unwinding duplex DNA while it synthesizes RNA primers for the lagging strand polymerase. DNA synthesis on both strands is catalyzed by a holoenzyme complex formed by the polymerase and a processivity β-clamp. The clamp is loaded onto the DNA by the clamp loader complex. The leading and lagging strand holoenzymes interact to form a dimer. Single-stranded DNA resulting from helicase activity is coated with single-stranded DNA-binding protein (SSB).

DNA replication in bacteria is performed by a large, multiprotein complex called the replisome, which synthesizes the leading and lagging strands in a highly coordinated manner. The replisome proteins catalyze a huge number of events, such as DNA unwinding, RNA primers synthesis, clamp loading and DNA polymerization. Comparative genomic analyses demonstrated that most of the replisome components are conserved across bacteria [20]; however, this assumption is not valid for MTB, which lacks obvious homologs of several components that perform key functions in model organisms such as *Escherichia coli* and *Bacillus subtilis*. As excellently reviewed in the paper of Ditse and co-authors [55], the bacterial replication machinery is based on the concerted action of three catalytic centers: the helicase-primase complex; the core complex; and the clamp loader complex (Figure 2).

### 3.1. The Helicase-Primase Complex

Although it is common to refer to the *Escherichia coli* model when considering the overall process of DNA replication in MTB, it is important to note that some notable differences among these species have been described, including the observation that MTB lacks clear homologs of several initiation proteins (DnaC, DnaT, PriB and PriC) [55]. The helicase-primase complex (primosome) mainly refers to the DnaB helicase, the DnaG primase and single-stranded DNA binding proteins (SSB) that constitute the basic replication module in bacterial genomes [20,56]. Despite the absence of a canonical helicase loader (DnaC in *Escherichia coli*), it was recently demonstrated that MTB possesses an ancestral bacterial replicative operator named DciA (Rv0004), which displayed the attributes of the replicative helicase-operating proteins associated with replication initiation [57,58]. Considering the key role of the helicase-primase complex in the series of events taking place in mycobacterial replication, it should be considered a valid drug target. However, the difficulties encountered in the in vitro reconstruction of such protein complexes have restricted the number of screenings against purified proteins, as well as of the structure-based drug design approach, which at present could finally refer to impressive progress of cryo-EM-based techniques [59]. Most of the drug discovery pipeline targeting the primosome focused on the identification of small-molecule inhibitors of SSB-protein interaction. SSB-protein plays a critical role in protecting unwound single-stranded DNA, and binds target DNA with high affinity and in a sequence-independent manner. In addition to DNA binding, SSB also physically associates with a number of different genome maintenance proteins [60]. Small molecules that disrupt *Escherichia coli* SSB interaction with the Exonuclease I binding partner have been identified [61,62], and were validated for their antibacterial activity against a diverse panel of bacterial species [63]. The DnaG primase synthesizes primers for lagging strand Okazaki fragments, and a number of inhibition studies aim at targeting bacterial primase. They identified natural products as attractive inhibitors of DnaG from *Escherichia coli* [64,65]. However, whole-cell activity was only considerable in a mutant strain deficient in the lipopolysaccharide layer, strengthening the role of the bacterial cell wall in the permeation and efflux of antibiotics. The primosome activity is coordinated and facilitated by the replicative DnaB helicase, and despite there being no published results on active molecules blocking the mycobacterial protein, some works reported a number of flavonols that have been shown to inhibit helicase activity in other bacterial species [66,67]. Interestingly, mycobacterial DnaB contains an intein motif whose splicing is blocked under oxidizing conditions, allowing the arrest of replication, which should be advantageous to preserve DNA integrity in the presence of reactive oxygen species [68]. While the drug discovery value of targeting proteins belonging to helicase-primase complex remains to be determined for MTB, these results support the potential of investigating the primosome as a novel anti-mycobacterial target. 

### 3.2. The Core Complex and the Clamp Loader Complex

At present, there are no anti-TB drugs in clinical use which directly target the replisome core complex in MTB; however, by adopting a drug-revisiting approach, Kling et al. validated a natural product called griselimycin (extracted from *Streptomyces* spp) as a potent inhibitor of the sliding β-clamp. In particular, they improved the griselimycin pharmacological properties by developing one synthetic derivative (cyclohexylgriselimycin) with a higher penetrating capacity in cells of the immune system that harbor the MTB bacilli. Moreover, in combination with other drugs, the griselimycin derivative showed high potency in mice with TB [69]. Cyclohexylgriselimycin binds with dissociation constant in the nanomolar range to the *dnaN*-encoded sliding β-clamp of MTB, without interfering with the human DNA clamp protein, PCNA, resulting in a very high selectivity index [69].

### 3.3. DNA Topology Control and Regulation

DNA unwinding by DNA gyrase and topoisomerase could be considered as a parallel molecular event directly linked to the replication of chromosomal DNA. Indeed, DNA topology control is functional to an efficient processive synthesis, as well as the elimination of the stresses resulting from negative supercoiling and the concatenation of double-stranded DNA. The type II topoisomerase, DNA gyrase, is built on two subunits, gyrase A and gyrase B, which together form the catalytically active heterotetrameric enzyme (i.e., GyrA_2_B_2_) [70]. The role of the A subunit is the breakage and rejoining of the double DNA strand, while the B subunit possesses the ATPase activity, which provides energy for the DNA supercoiling. MTB lacks topoisomerase IV, and the negative supercoiling of DNA is performed only by gyrase (18). The enzyme is a clinically validated drug target, and the currently available inhibitors can be classified based on their origin, i.e., drugs obtained from natural sources or synthetic drugs. Among the latter group, fluoroquinolones represent the most successful antibacterial agents targeting DNA gyrase; these compounds have been extensively explored to improve the spectrum of activity and potency, and are currently used as second-line anti-TB agents. The mechanism of the action of fluoroquinolones involves the stabilization of the covalent gyrase A subunit-DNA complex, thereby leading to protein-stabilized DNA breaks with a bactericidal outcome [71]. Aminocoumarins such as novobiocin are natural products, and they inhibit the ATPase function of the B subunit [72], which appears less exposed to mutagenic events compared to the A subunit [73]. However, the relatively poor pharmacokinetic profile of this class of natural compounds limited their use in clinics as an anti-TB drug. In the context of DNA topology modulation, type I topoisomerase TopA (Rv3646c), which causes single-stranded nicks in relaxing the DNA, is inhibited by hydroxycamptothecin, a derivative of the anticancer topoisomerase inhibitor camptothecin (18). The functional modification of camptothecin scaffolds led to the discovery of whole-cell activity on both drug-sensitive and XDR MTB strains [74,75], validating MTB TopA as an innovative drug target to be considered in further medicinal chemistry studies. Finally, Okazaki fragments generated during the replication process are ligated by the mycobacterial NAD^+^-dependent DNA ligase (Rv3014c), which is therefore considered essential, and was deeply investigated as a drug target. However, although several in vitro inhibition studies on recombinant MTB LigA are present in scientific literature, very few molecules have been shown to exhibit whole-cell activity in the micromolar range against MTB [76,77,78,79].

## 4. Targeting MTB DNA Repair

During its entire life cycle, MTB must face a multitude of DNA-damaging stresses and a continuous exposure to harmful agents, which could compromise bacterial fitness as the result of increased genomic instability. The relative contribution of different DNA repair activities to the maintenance of MTB chromosome stability at different stages of the infection has been exhaustively reviewed [80]. In particular, one of the hallmarks of MTB infection is the bacilli’s ability to survive the hostile environment of the host’s infected macrophages, and the release of chemicals by the host cells that lead to bacterial DNA damage, mainly induced by endogenous DNA-alkylating agents originated by the action of highly reactive oxidative (ROS) and nitrosative (RNI) radicals [81,82]. The reaction of nitrogen monoxide radicals with oxygen produces nitrous anhydride, which nitrosates amines and amides to produce compounds that are converted by a metabolic pathway into potent DNA alkylating agents. Significant progress has been made in the knowledge of bacterial physiology as result of in-depth genomic analyses and gene inactivation studies. Aside from the notable exception of canonical Mismatch Repair (MMR) components, the DNA damage response of MTB includes most of the DNA repair pathways described in other bacterial species: (i) multi-enzymatic systems like Nucleotide Excision Repair (NER) and Base Excision Repair (BER); (ii) recombination repair systems; and (iii) single proteins responsible for the direct reversal of DNA damage [83]. Interestingly, a NucS-dependent DNA repair system that potentially replaces the MutS/MutL-based MMR was recently identified in *Mycobacterium smegmatis* [84]. DNA repair pathways should be considered an attractive source of drug targets, since they provide essential functions to the bacteria [45], and in many cases, the enzymatic cascade requires proteins that are distinct from the human ones at the biochemical and structural level, potentially ensuring drug selectivity. Oxidative and pro-alkylation stresses are mainly counteracted by the action of the multi-step NER pathway, in which the damage recognition and subsequent endonucleolytic reactions are carried out by the coordinated action of the UvrA, UvrB and UvrC proteins [85]. In MTB, as observed in other bacteria, the NER cascade begins with formation of a macromolecular complex between UvrA and UvrB proteins that were structurally and biochemically characterized [86,87]. It was demonstrated that the two proteins interact in solutions in the absence of ligands, supporting the hypothesis that the scouting of damaged sites inside DNA could involve pre-assembled UvrA_2_/UvrB_2_ heterotetramers, and suggesting the possibility of targeting UvrA-UvrB in order to block the entire NER cascade in MTB [88,89]. Moreover, an inhibitor of the endonuclease activity of the UvrABC complex [2-(5-amino-1,3,4-thiadiazol-2-ylbenzo[f]chromen-3-one) (ATBC)] was identified, which was active at a micromolar concentration [90]. However, the mechanistic aspects of ATBC inhibitory activity and the direct target inside the UvrABC system are still unknown. The full complement of genes that encode for homologs involved in the BER pathway have been detected in MTB [5]. Despite proteins belonging to BER pathways in mycobacteria being highly conserved, they have not yet been exploited for the design of new compounds that target this pathway. As an alternative to the multi-enzymatic DNA repair response, MTB also counteracts the deleterious effect of alkylating agents by the expression of inducible genes of Ada response [91,92]. The domains of Ada (i.e., AdaA and AdaB), AlkA and AlkB proteins exist in different combinations in different bacteria. In particular, MTB shows a gene fusion of adaA with alkA (AdaA-AlkA), and an independent *adaB* gene, also annotated as *ogt*, which encodes for the OGT protein (Rv1316c). MTB OGT, as the orthologous proteins of other organisms, invariably performs the removal of alkyl adducts on modified guanines through a suicidal mechanism, by catalyzing the stoichiometric transfer of the O6-alkyl group to the strictly conserved cysteine residue in the protein active site, which is hosted in the C-terminal domain [93,94,95]. The protein’s overall structure and mechanistic aspects of the suicidal reaction are highly conserved among prokaryotic OGTs, as well as in the human equivalent enzyme [96,97,98,99,100], which is a validated target for cancer chemotherapy [101]. Small-molecule inhibitors of the human enzyme, which are currently used as adjuvants in antineoplastic therapeutic regimens, have not been tested on MTB; indeed, their potential exploitability as anti-tubercular drugs is limited by their cross-reactivity with the human protein, possibly resulting in a genotoxic effect. While nucleotide synthesis and DNA replication have both been exploited for the development of antibacterial agents and are therefore logical and solid targets for the discovery of novel antitubercular drugs, DNA repair is proposed as a new target for the design of drugs with a completely new mechanism of action, and therefore potentially active against drug-resistant strains. However, this poses significant challenges not only for selectivity of drug action and drug resistance, but also for a potential risk of emergence of hypervirulent bacteria. Indeed, as the DNA repair is a major mechanism limiting the occurrence of spontaneous and/or induced mutations, its inactivation could result in hypermutability with the selection of a hypervirulent strain.

## 5. Conclusions

A number of biochemical events ensure the genome integrity in all organisms; many of these are highly conserved and essential. Moreover, most of the protein complexes active in the maintenance of DNA homeostasis in bacteria, including MTB, are distinct from those in eukaryotes, and should be considered a valid source of molecular targets for antibiotic design and development. To this end, we summarize in this review current understanding of biochemical and structural features of mycobacterial DNA metabolism pathways, and particularly focused on the three main processes described below: (i) synthesis of nucleotide precursors, describing the drug discovery approaches targeting purine and pyrimidine biosynthesis at level of the key enzymatic steps; (ii) DNA synthesis, analyzing the components of replication machinery (i.e., the helicase-primase complex, the core complex and the clamp loader complex) that have been exploited for the development of antibacterial agents; and (iii) DNA repair and damage reversal, widely unexploited as a drug target, discussing the innovative strategies that have been suggested to interfere with mycobacterial responses to DNA damage. The classes of approved drugs targeting DNA metabolism, along with those that have been characterized in preliminary in vitro studies, described in the manuscript, are listed in Table 1.

## Figures and Tables

**Table 1:** Essential proteins involved in DNA metabolism targeted by anti-bacterial compounds.

Target Protein	In Vitro Essentiality [30,45]	Inhibitor Molecules/Classes
**GuaB2**	Essential	diphenyl urea derivatives: DPU-2, DPU-3 [29]
		triazole-linked mycophenolic adenine [33]
		indazole sulfonamides [36]
		VCC234718 5-(4-cyclohexanecarbonylpiperazine-1-sulfonyl)isoquinoline [37]
		5-amidophthalide derivative [38]
		Benzoxazole derivatives [39]
**OPRT**	Essential	Hydroxy-2-oxo-1,2-dihydropyridine-4-carboxylic acid and its derivative 3-Benzylidene-2,6-dioxo-1,2,3,6-tetrahydropyridine-4-carboxylic acid [48]
**SSB (*E.coli*)**	Essential	Small-molecule inhibitors ^1^ [61]
**DnaG (*E.coli*)**	Essential	Phenolic monosaccharides ^1^ [64]
		Bicyclic macrolide ^1^ [65]
**DnaB (*E.coli, K. Pneumoniae*)**	Essential	Flavonols ^1^ [66,67]
**DnaN/β**	Essential	Griselimycins [69]
**GyrA**	Essential	Quinolones [71]
**GyrB**	Essential	Novobiocin and coumarin derivatives [72,73]
**TopA**	Essential	m-AMSA [74]
		Norclomipramine and Imipramin [75]
		Hydroxycamptothecin derivatives [18]
**LigA**	Essential	Pyridochromanone [76]
		Bis-xylofuranosylated diamines [77,79]
		N-substituted tetracyclic indoles [78]
**UvrABC complex**	Essential	2-(5-amino-1,3,4-thiadiazol-2-ylbenzo[f]chromen-3-one) (ATBC) [90]

^1^ The compounds refer to inhibition studies performed on homologs of essential MTB proteins belonging to other bacterial species, indicated in brackets.
